# A clinical practical model for preoperative prediction of visual outcome for pituitary adenoma patients in a retrospective and prospective study

**DOI:** 10.3389/fendo.2024.1479442

**Published:** 2024-12-13

**Authors:** Zijian Zheng, He Wang, Qianxi Chen, Zhicheng Wang, Jun Fu, Wenjian Fan, Yuanxiang Lin, Dezhi Kang, Changzhen Jiang, Zhangya Lin, Xiaorong Yan

**Affiliations:** ^1^ Department of Neurosurgery, National Regional Medical Center, Binhai Campus of the First Affiliated Hospital, Fujian Medical University, Fuzhou, Fujian, China; ^2^ Department of Neurosurgery, Neurosurgery Research Institute, The First Affiliated Hospital, Fujian Medical University, Fuzhou, Fujian, China; ^3^ Department of Neurosurgery, Xuanwu Hospital, Capital Medical University, China International Neuroscience Institute, Beijing, China; ^4^ Fujian Provincial Institutes of Brain Disorders and Brain Sciences, First Affiliated Hospital, Fujian Medical University, Fuzhou, Fujian, China

**Keywords:** pituitary adenoma, clinical practical model, visual outcome, graphic segmentation, machine learning (ML)

## Abstract

**Objective:**

Preoperative prediction of visual recovery after pituitary adenoma resection surgery remains challenging. This study aimed to investigate the value of clinical and radiological features in preoperatively predicting visual outcomes after surgery.

**Methods:**

Patients undergoing endoscopic transsphenoidal surgery (ETS) for pituitary adenoma were included in this retrospective and prospective study. Preoperative MRI, visual acuity, visual field, and postoperative visual recovery data were collected. Logistic regression analysis was used to assess the importance of clinical and MRI features, and a prediction model was developed.

**Results:**

The cohort included 198 patients (150 retrospective, 48 prospective). In the retrospective data, visual recovery was observed in 111 patients (74.0%), while non-recovery was observed in 39 patients (26.0%). In the prospective data, visual recovery was observed in 27 patients (56.25%) and non-recovery in 21 patients (43.75%). Blindness, headache, adenoma area, and adenoma upward growth distance were negatively correlated with visual recovery (p < 0.05), while the pituitary gland area was positively correlated (p = 0.001). Logistic regression selected three clinical features: blindness, headache, and visual impairment course. Two additional imaging features, pituitary gland maximum area, and adenoma maximum area, were incorporated into the prediction model. The area under the curve of the prediction model was 0.944 in the retrospective cohort and 0.857 in the prospective cohort. Accuracy was 88% and 81.25%, respectively.

**Conclusion:**

This study successfully developed a clinical practical model combining clinical and radiological features to preoperatively predict visual recovery for patients with pituitary adenoma. The model has the potential to provide personalized counseling for individual patients.

## 1 Introduction

Pituitary adenoma constitutes the most prevalent types of central nervous system adenoma, comprising 12-15% of intracranial adenoma (1). These adenomas can manifest as endocrine abnormalities, visual impairment, headache, and cognitive dysfunction. Visual impairment, arising from the adenoma’s characteristic upward growth and compression of the optic chiasma, stands out as the predominant symptom, reported by 32% to 70% of patients (2). The primary manifestations of visual impairment encompass reduced visual acuity (VA) and defects in the visual field (VF) (3).

Endoscopic transsphenoidal surgery (ETS) represents the principal method for alleviating symptoms resulting from adenoma compression, facilitating visual recovery in patients with secondary visual impairment due to pituitary adenoma. Existing studies have indicated that factors such as patient age, adenoma size, symptom course, and preoperative visual field defects significantly impact the postoperative recovery of visual impairment (4–8). Despite these findings, a preoperative clinical solution capable of predicting postoperative visual acuity recovery and quantifying the extent of postoperative recovery remains a challenge.

Prior research has proposed that evaluating the extent of upward extension of pituitary adenoma through magnetic resonance imaging (MRI) could serve as a predictor for the degree of visual impairment in individuals with non-functioning pituitary adenoma (NFPA) featuring optic chiasm compression (9). Additionally, Optical coherence tomography (OCT) was used to measure the thickness of the retinal nerve fiber layer (RNFL), offering a method to forecast the extent of postoperative visual recovery in patients (10). Subsequently, Li et al. employed OCT to measure macular ganglion cell-inner plexiform layer thickness (mGCIPL), a direct indicator of ganglion cell injury severity, aiming to predict postoperative visual recovery (11, 12). Furthermore, there has been exploration into the use of optic chiasma analysis on conventional MR imaging for preoperative prediction of visual recovery following surgical decompression. It is worth noting, however, that the model exhibited limitations in predicting outcomes for patients with severe compression of the optic chiasm, especially for patients with invisible optic chiasm on preoperative MRI (13).

Previous studies have indicated that the recovery of patients’ visual acuity after surgery is intricately linked to multiple factors (14). Consequently, relying on a single factor alone may not provide a comprehensive explanation of patient prognosis. Presently, model construction based on the assignment of weights to multiple factors offers the potential for achieving more accurate clinical outcomes in a convenient and expeditious manner. Earlier investigations have demonstrated that integrating imaging data with clinical features can enhance the preoperative diagnosis and prognosis of pituitary adenoma (15).

In this study, we retrospectively included 150 patients and conducted an analysis of the correlation between preoperative clinical features and imaging characteristics and the recovery of postoperative visual acuity. We developed a model based on preoperative clinical features and imaging characteristics. To validate the preoperative predictive capability of the model for visual prognosis, we prospectively included 48 patients.

## 2 Methods

### 2.1 patients

In this study, we conducted a retrospective review of 150 patients presenting with visual impairment due to PAs who were treated at the First Affiliated Hospital of Fujian Medical University between January 2018 and December 2022. Additionally, we prospectively analyzed 48 patients with pituitary adenoma-related visual impairment treated at the same hospital from January 2023 to June 2023. Comprehensive medical records were available for all cases before surgery, encompassing details such as age, gender, initial symptoms, symptom course, ophthalmic evaluation, pituitary gland MRI, and postoperative pathology reports.

The inclusion criteria included: 1) Patients diagnosed with PAs who underwent endoscopic transsphenoidal surgery (ETS). 2) Absence of other causes of vision loss, as confirmed by ophthalmological assessment. 3) MRI evidence indicating optic nerve compression. 4) Availability of complete postoperative follow-up data.

Exclusion criteria included: 1) Prior history of anterior, posterior, or optic nerve diseases (excluding compressive optic neuropathy), such as glaucoma, local incision, or optic nerve hemorrhage. 2) Exclusion of patients with unreliable preoperative visual field testing.

### 2.2 Image data

**Figure 1:**
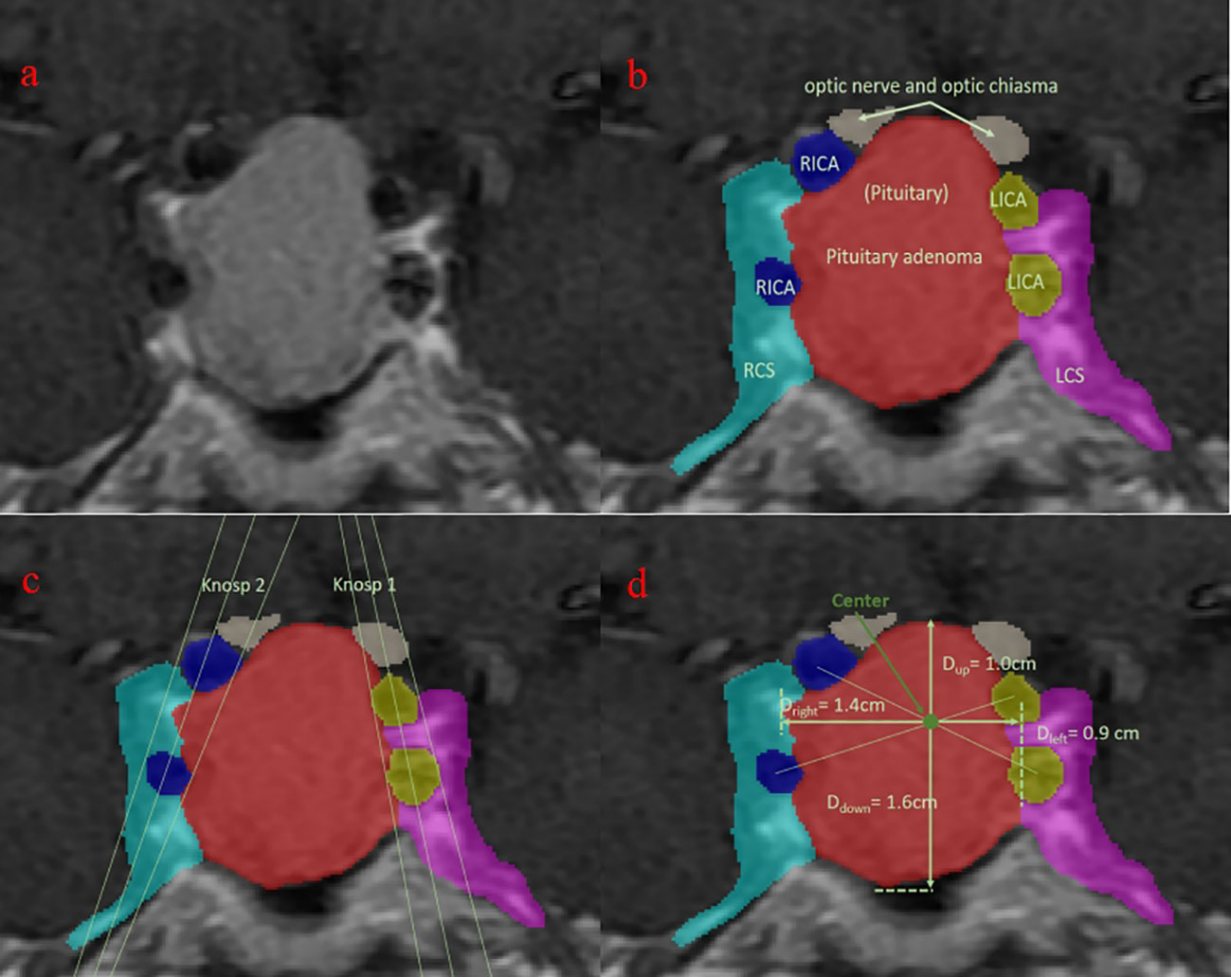
Visualization of the segmentation. Blue, right internal carotid artery; green: normal pituitary (not seen); light blue: right cavernous sinus; purple, left cavernous sinus; red: pituitary adenoma; white, optic nerve and optic chiasm; yellow, left internal carotid artery. **(A)** original MRI slice with the largest tumor area; **(B)** segmentation of different tissues; **(C)** Knosp grade based on the segmentation; **(D)** upward growth distance of pituitary adenoma.

Sellar region MR images were conducted on all patients through pituitary gland scanning utilizing 1.5T and 3.0T MRI scanners. The scanning protocol comprised T1-weighted images (T1WI), T2-weighted images (T2WI), and T1-enhanced sequences, each with a thickness of 3 mm. The obtained images were subjected to automatic segmentation using deep learning on coronal images, classifying them into eight categories: background, pituitary adenoma (PA), normal pituitary gland, right internal carotid artery (ICA), right cavernous sinus (CS), left ICA, left CS, and optic chiasm (OC) (**Figure 1**) (16). Subsequently, the segmentation results were manually reviewed and modified by two senior neurosurgeons with 10 years of experience.

### 2.3 Ophthalmic evaluation

A comprehensive review of clinical data for all patients included an in-depth analysis of the disease course and the visual impairment course. Patients underwent a thorough assessment of visual function, utilizing LogMAR visual acuity charts and automated perimetry. Postoperatively, patients were closely monitored for improvements in visual function, assessing whether there was improvement, complete improvement, and the time intervals for such improvements.

### 2.4 Feature collection

#### 2.4.1 Clinical characteristics

Recording the presence of symptoms such as headache, blindness, gender, age, BMI, visual impairment course, and disease course.

#### 2.4.2 Image characteristics

Including the maximum adenoma area and pituitary gland area across all slices, upward growth distance of pituitary adenoma, the optic chiasm (or optic nerve) area in the largest adenoma area slice, and Knosp grade.

### 2.5 Follow-up details

A telephone-based follow-up survey was conducted for each patient, addressing the following questions:

- Whether there was preoperative visual impairment (visual field defect, decreased visual acuity).
- Postoperative recovery of visual impairment.
- The degree of recovery, categorized as complete recovery or recovery with residual visual impairment.
- Time to improvement, specifying whether improvement was immediate or occurred after a certain period.

Based on the responses to the follow-up questions, patients were categorized into two groups:

1. Non-recovery Group: Patients whose visual impairment either persisted or worsened before or after surgery.
2. recovery Group: Patients whose visual impairment partially recovered or returned to normal after surgery.

In addition, before ETS and approximately 1week and 1 week after surgery, all patients were completed VF-14 (details for **Supplementary Materials**).

### 2.6 The construction of the clinical model

We initially calculate the cutoff value based on the distribution of each dataset and discretize each variable accordingly. Subsequently, univariate and multivariate analyses are employed to identify the relevant variables to be included in the model construction. The model framework is then established by integrating clinical prior knowledge. Finally, logistic regression is applied to calculate the weight assigned to each variable, completing the construction of the clinical prognostic model.

### 2.7 Statistics

Analyses were conducted using SPSS 26.0 and Graphpad Prism statistical software. Normally distributed measurement data are presented as $\bar{\text{x}}\pm \text{s}$ , skewed distribution measurement data as M (range), and enumeration data as absolute numbers. Depending on the distribution pattern, continuous normally distributed variables were compared using t-tests while continuous non-normally distributed variables were compared using non-parametric tests. Multiple linear regression analysis was employed to identify potential risk factors (MD values) for visual impairment, with the significance level set at P < 0.05.

## 3 Results

### 3.1 Patient clinical characteristics

**Figure 2:**
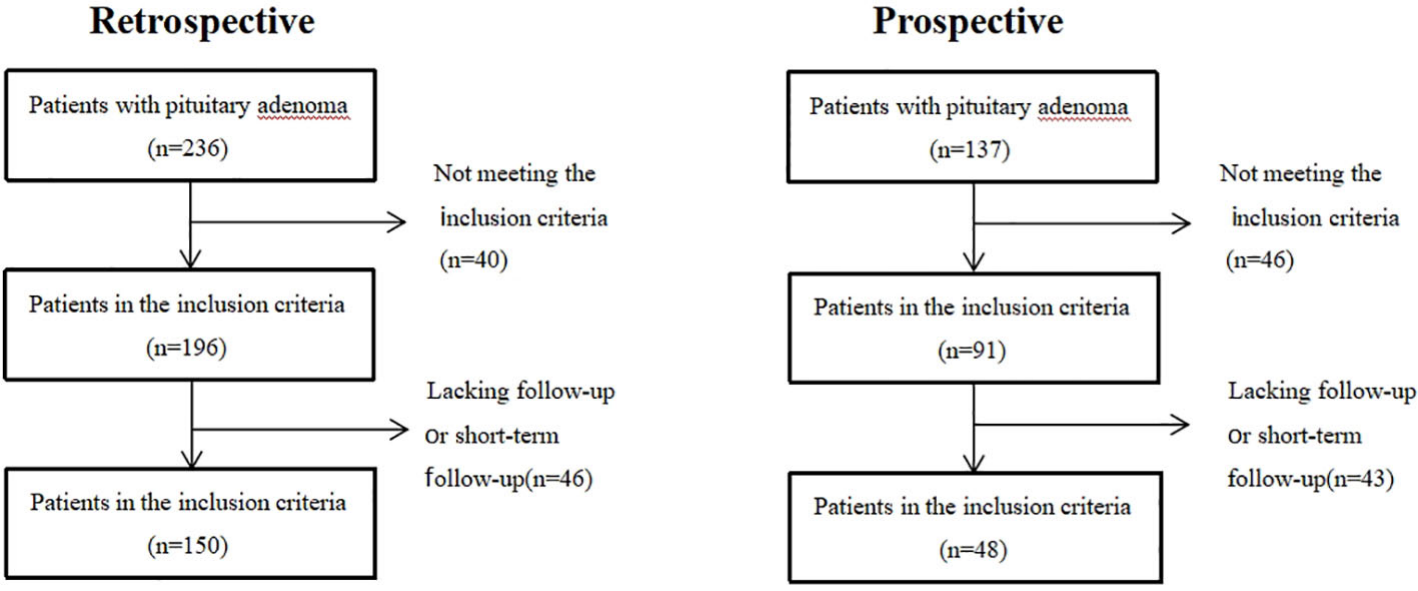
Flowchart of the retrospective and prospective datasets.

A total of 198 patients with PAs (including 150 retrospective patients and 48 prospective patients) were enrolled in this study (**Figure 2**). Among the retrospective patients, 111 (74%) experienced visual recovery following pituitary adenoma surgery and were classified as the recovery group, while 39 (26%) remained visually impaired after surgery and were classified as the non-recovery group. Patients who were blind will not be restored after operation(p = 0.001). Preoperative headache (p = 0.001), adenoma area (p = 0.001), and upward growth distance of pituitary adenoma (p = 0.001) were negatively correlated with postoperative visual recovery, while patients’ pituitary gland area (p = 0.001) was positively correlated with postoperative visual recovery. Among prospective patients, 27(56.25%) were classified as the recovery group, while 21(43.75%) were classified as the non-recovery group (**Table 1**).

**Table 1:** Patient clinical characteristics.

	Retrospective dataset			Prospective dataset		
	Recovery	Non-recovery	*p*	Recovery	Non-recovery	*p*
Number	111	39		27	21	
Blindness	0 (0%)	7 (18%)	**0.001**	0 (0%)	5 (23.8%)	**0.007**
Headache	25 (22.5%)	24 (61.5%)	**0.001**	10 (37.0%)	9 (42.9%)	0.516
Sex (female)	53 (47.7%)	25 (64.1%)	0.078	12 (44.4%)	8 (38.1%)	0.658
Age (years)	51.3 ± 12.0	52.5 ± 14.8	0.623	43.56 ± 13.3	46.86 ± 15.5	0.086
BMI	24.1 ± 3.06	24.5 ± 2.42	0.470	23 (20.2-27.7)	24 (21-40.3)	0.851
Course (month)	6 (0.5-36)	12 (0.5-36)	0.860	4 (0.2-36)	12 (0.25-36)	0.448
Eye course (month)	3 (0.25-24)	10 (0.5-24)	0.178	2 (0.5-12)	12 (0.25-36)	0.059
MRI						
Adenoma (mm^2^)	633 (215-1538)	824 (244-1527)	**0.001**	666 ± 242	869 ± 435	**0.009**
Pituitary gland (mm^2^)	25.78 (0-74.15)	2.40 (0-22.65)	**0.001**	44.75 (0-89)	23.5 (0-80)	**0.012**
Up max (mm)	21.4 ± 6.4	27.7 ± 7.8	**0.001**	18 ± 10.2	19.0 ± 11.4	0.098
OC (mm^2^)	0.68 (0-61.08)	0 (0-48.06)	0.100	2 (0-60.75)	10.75 (0-80)	0.564
Knosp grade			0.749			0.823
0	53	14		3	4	
1	19	10		5	6	
2	17	5		10	5	
3a	11	5		2	1	
3b	8	4		4	2	
4	3	1		3	3	

Course, The duration of patient’s illness. Eye course, The course of visual impairment.

Adenoma, The maximum adenoma area across all slices. Pituitary gland, The maximum pituitary gland area across all slices.

Up max, Upward growth distance of pituitary adenoma. OC, the optic chiasm (or optic nerve) area in the largest adenoma area slice.

The bold values indicate that the p-value for this feature is less than 0.05, highlighting its statistical significance.

### 3.2 Cutoffs of contributing factors

**Figure 3:**
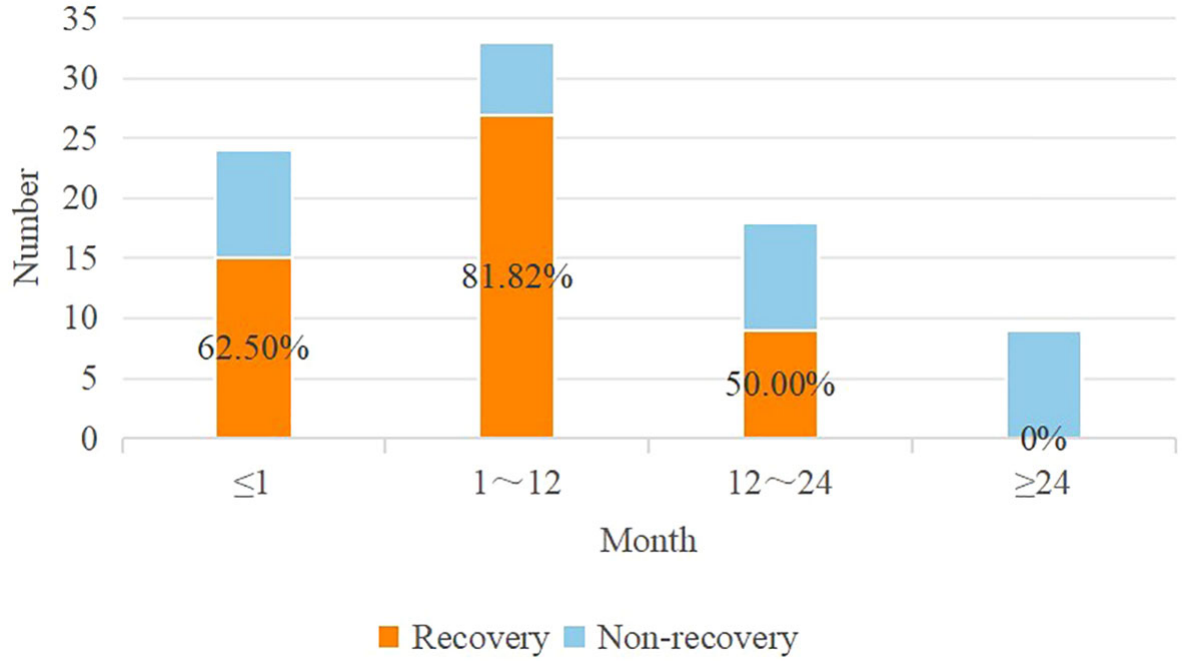
Distribution of the visual impairment course in the retrospective dataset.

Cutoff values were identified using AUC curves in the retrospective cohort (**Table 2**). Based on these cutoff values, continuous variables in the table were discretized. When examining the AUC curve for the visual impairment course, it was found that a single cutoff value could not be applied. Therefore, patients were divided into the following groups (0 ~ 1 month, 1 ~ 12 months, 12 ~ 24 months, and more than 24 months), shown in **Figure 3**. Simultaneously, it was observed that visual acuity was recovery after surgery when the patient’s pituitary gland maximum area was greater than 25 mm^2^.

**Table 2:** Cutoffs of features.

	Retrospective dataset			
	AUC	Sensitivity	Specificity	Cut off
Age (years)	0.527	0.351	0.846	56.500
BMI	0.541	0.462	0.84	24.696
Adenoma (mm^2^)	0.688	0.703	0.692	750.372
Pituitary gland (mm^2^)	0.742	0.541	1	25.000
Up max (mm)	0.732	0.973	0.385	30.811
OC (mm^2^)	0.640	0.514	0.846	0.515

Adenoma, The maximum adenoma area across all slices. Pituitary gland, The maximum pituitary gland area across all slices.

Up max, Upward growth distance of pituitary adenoma. OC, the optic chiasm (or optic nerve) area in the largest adenoma area slice.

### 3.3 Factors affecting postoperative mean deviation

Logistic multivariate analysis indicated that upward adenoma growth (OR, 0.902; 95% CI, 0.116-7.021; p = 0.921) was not significantly associated with postoperative visual acuity recovery. Multivariate analysis revealed that preoperative headache (OR, 6.754; 95% CI, 2.006-22.738; p = 0.002), longer duration of preoperative visual impairment (OR, 0.095; 95% CI, 0.019-0.473; p = 0.004), and larger adenoma area (OR, 0.101, 95% CI, 0.012-0.885; p = 0.038) were associated with poorer postoperative visual recovery (**Table 3**).

**Table 3:** Univariate and multivariate analyses measure the correlation between the clinical features and visual remission.

Variable	Univariate analysis			Multivariate analysis		
	OR	95%CI	P value	OR	95%CI	P value
Headache	5.687	2.178-14.855	0.001	6.754	2.006-22.738	0.002
Eye course	0.170	0.068-0.422	0.001	0.095	0.019-0.473	0.004
Adenoma	0.238	0.094-0.604	0.003	0.101	0.012-0.885	0.038
Up max	0.233	0.077-0.707	0.010	0.902	0.116-7.021	0.921

Eye course, Visual impairment course. Adenoma, The maximum adenoma area across all slices. Up max, Upward growth distance of pituitary adenoma.

### 3.4 Construction of predictive model

**Figure 4:**
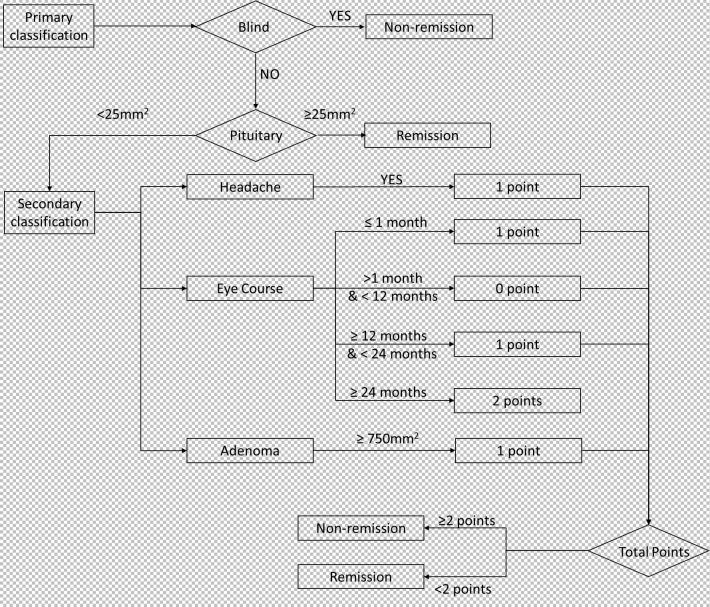
The proposed model to preoperatively prediction of the visual outcome.

We observed that postoperative visual acuity did not recover in blind patients (**Table 1**). And according to data from follow-up, we founded when the patient’s pituitary gland maximum area exceeded 25 mm^2^, postoperative visual acuity was recovered. Then, based on multivariate logistic regression, three factors (headache, eye course, and adenoma area) were identified as key factors. Patients who with headaches and a adenoma area greater than 750 mm^2^, recovered worse postoperatively, so a score of 1 was assigned for these patients. Poor postoperative visual recovery was noted when the course of visual impairment was less than 1 month or exceeded 12 months, and recovery was worse when the course exceeded 24 months. Therefore, a score of 1 was assigned for patients with a course of visual impairment less than 1 month (including 1 month) or more than 12 months (including 12 months) and less than 24 months, and a score of 2 for those with a course exceeding 24 months (including 24 months). Postoperative visual acuity recovery was observed when the total score was less than 2. Subsequently, we constructed a predictive model (**Figure 4**).

### 3.5 Performance of the model in prospective patients

**Figure 5:**
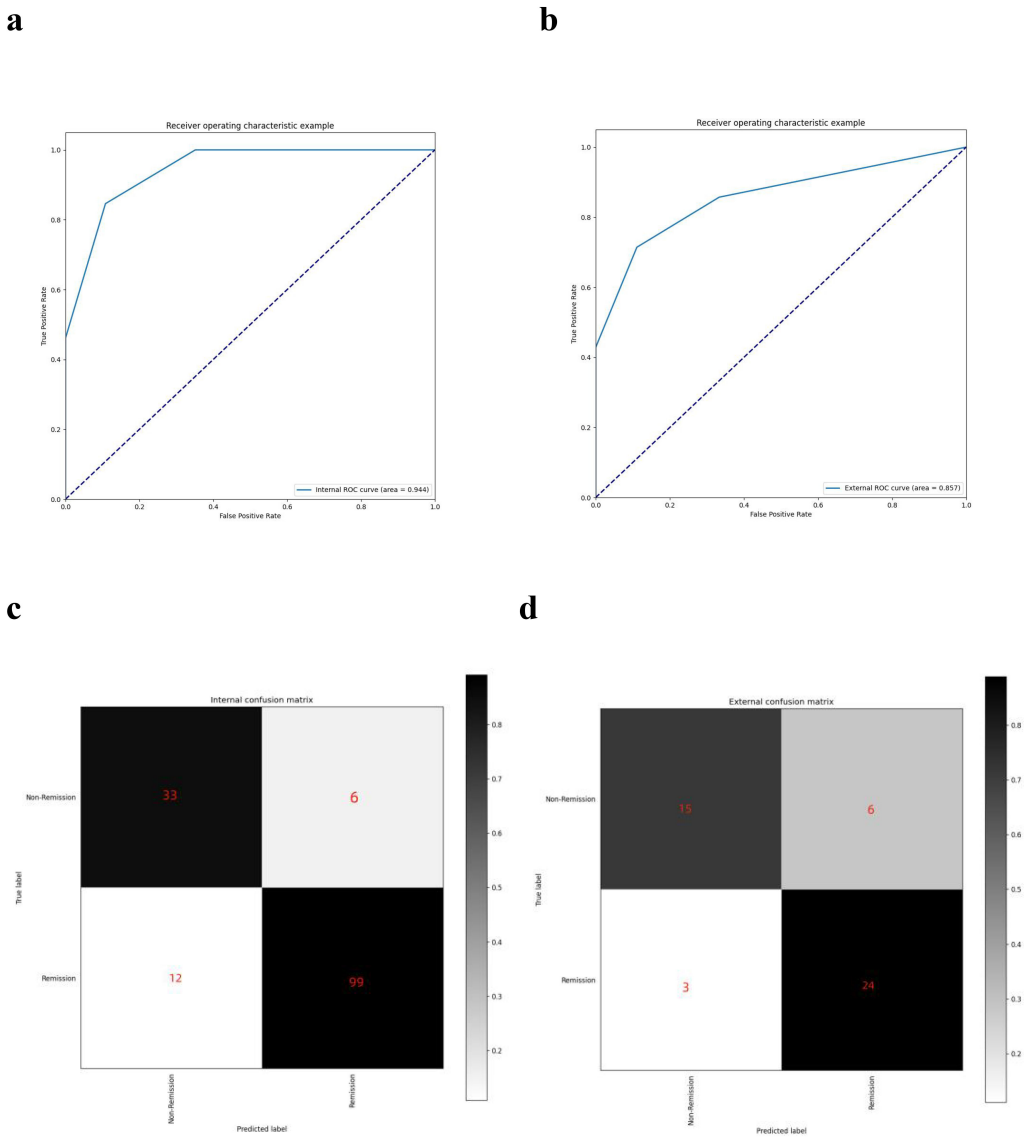
AUC curves and confusion matrixes. **(A)** the AUC curve of the model in the retrospective dataset; **(B)** the AUC curve of the model in the prospective dataset; **(C)** the confusion matrix of the model in the retrospective dataset; **(D)** the confusion matrix of the model in the prospective dataset.

The model, including its assignments, was established based on a retrospective cohort and then validated in a prospective cohort. As depicted in **Figure 5**, the AUC was 0.944 for the retrospective cohort and 0.857 for the prospective set cohort. The AUC values exceeding 0.85 indicate that the model can predict visual function recovery preoperatively. The confusion matrix in the retrospective and prospective validation cohorts is illustrated in **Figure 5**. The accuracy was 88% in the retrospective cohort and 81.25% in the prospective cohort, with most cases correctly classified.

## 4 Discussion

The pituitary adenoma, a common primary central nervous system adenoma, often presents with visual dysfunction in 32% to 70% of patients, making it a crucial surgical indication (5). The restoration of visual function is vital for surgical decision-making and doctor-patient communication. Previous studies have predominantly focused on factors associated with the occurrence of preoperative visual impairment, such as patient age, adenoma area, visual impairment course, and specific preoperative visual field defects (4–8). However, limited attention has been given to factors influencing visual recovery after surgery. This study extensively explored factors related to postoperative visual disturbance recovery, including pituitary gland area and the distance of upward growth of pituitary adenoma.

Qiao built a model for predicting visual recovery based on machine learning, achieving an AUC of 0.843 and an accuracy of 0.850. However, the construction of this model involved blood tests, OCT, MRI and relied heavily on machine learning algorithms, which significantly impacted its clinical practicality. The study constructed a clinically practical predictive model for visual recovery by incorporating clinical and imaging features, without the facilitating of computer programming and OCT examination. Key factors considered in the model were preoperative blindness, headache, vision impairment course, pituitary gland maximum size, and adenoma maximum size. The model demonstrated reasonable accuracy in both the retrospective (AUC = 0.944, ACC = 0.88) and prospective (AUC = 0.857, ACC = 0.8125) cohorts, which was comparable to the previous research but with rapid prognosis prediction and more clinical practical situations. Notably, preoperative blindness and small pituitary gland areas were identified as crucial determinants for postoperative unrecovered visual disturbances. Moreover, the visual impairment course is more important than the disease course. The scoring system derived from the model suggested that patients with a total score greater than or equal to 2 were unlikely to experience postoperative visual recovery.

**Figure 6:**
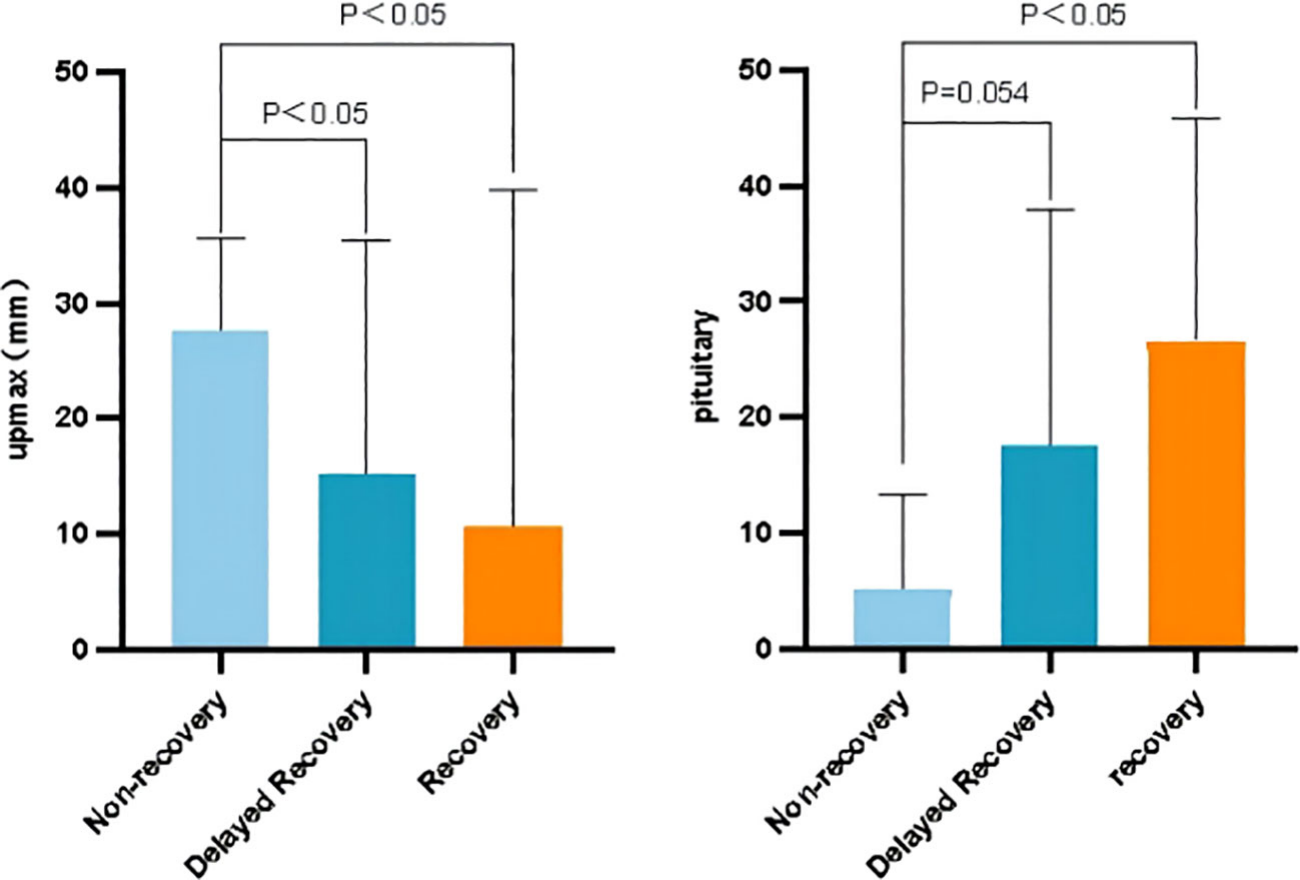
Patient clinical characteristics in subdivided datasets.

To further explore factors related to the visual recovery, we introduced sub-population classifications, distinguishing patients into non-recovery, delayed recovery, and immediate recovery groups. We defined the delayed recovery group as postoperative visual impairment recovered after more than one week, and defined the immediate recovery group as visual impairment returned to normal within one week after surgery. As **Figure 6** showed, we found patients with a shorter distance of upward adenoma growth recovered better postoperatively, and patients who with larger pituitary gland areas recovered better postoperatively.

While previous studies have noted the impact of pituitary macroadenoma on visual impairment and hypopituitarism, few have explored whether pituitary gland function, particularly hormone secretion, influences vision recovery (17–19). The current study revealed that the pituitary gland area is a significant influencing factor, with visual disturbances more likely to recover when the pituitary gland size exceeds 25 mm^2^, indicating the pituitary gland function is important for the recovery of the visual impairment. Interestingly, previous study has shown that pituitary-related hormone, such as total thyroxine, has a strong relation with visual impairment recovery, emphasizing the vital place of endocrinal function in prognosis (15). Moreover, our study highlighted the impact of clinical symptoms rather than findings in imaging results.

Several limitations were acknowledged. The study is a single-center study, resulting in potential selection bias and the small cohort size in the prospective study, hindering the construction of predictive models for sub-classification. Exclusion from loss to follow-up is somewhat large amount, which can lead to bias in developing a prognostic model. Future endeavors will involve multicenter studies, expanded sample collections for prospective analysis, refined sub-population classification predictions, and further exploration of the correlation between pituitary gland function and postoperative visual acuity recovery.

## 5 Conclusion

In summary, our study identified correlations between preoperative blindness, headache, adenoma maximum area, pituitary gland maximum area, and the distance of adenoma upward growth with postoperative visual recovery. In addition to, notably, patients with a shorter visual impairment course, shorter distance of tumor upward growth, and larger pituitary gland areas demonstrated better postoperative recovery. Furthermore, we developed a clinical practical model incorporating both clinical and radiological features that successfully predicts the likelihood of postoperative visual impairment recovery in pituitary adenoma patients. The model exhibited an accuracy of 0.88 and AUC of 0.944 in the retrospective study and an accuracy of 0.8125 and AUC of 0. 857 in the prospective study, offering a valuable tool for personalized counseling and treatment decisions for individual patients.

## Data Availability

The raw data supporting the conclusions of this article will be made available by the authors, without undue reservation.
