# Corrigendum: A clinical practical model for preoperative prediction of visual outcome for pituitary adenoma patients in a retrospective and prospective study

**DOI:** 10.3389/fendo.2024.1546763

**Published:** 2025-01-28

**Authors:** Zijian Zheng, He Wang, Qianxi Chen, Zhicheng Wang, Jun Fu, Wenjian Fan, Yuanxiang Lin, Dezhi Kang, Changzhen Jiang, Zhangya Lin, Xiaorong Yan

**Affiliations:** ^1^ Department of Neurosurgery, National Regional Medical Center, Binhai Campus of the First Affiliated Hospital, Fujian Medical University, Fuzhou, Fujian, China; ^2^ Department of Neurosurgery, Neurosurgery Research Institute, The First Affiliated Hospital, Fujian Medical University, Fuzhou, Fujian, China; ^3^ Department of Neurosurgery, Xuanwu Hospital, Capital Medical University, China International Neuroscience Institute, Beijing, China; ^4^ Fujian Provincial Institutes of Brain Disorders and Brain Sciences, First Affiliated Hospital, Fujian Medical University, Fuzhou, Fujian, China

**Keywords:** pituitary adenoma, clinical practical model, visual outcome, graphic segmentation, machine learning (ML)

In the published article, there was an error in affiliation 1. Instead of “Department of Neurosurgery, Binhai Branch of Nation al Regional Medical Center, The First Affiliated Hospital, Fujian Medical University, Fuzhou, Fujian, China”, it should be “Department of Neurosurgery, National Regional Medical Center, Binhai Campus of the First Affiliated Hospital, Fujian Medical University, Fuzhou, Fujian, China.”

There was another error in affiliation 4. Instead of “Department of Fujian Provincial Institutes of Brain Disorders and Brain Sciences, First Affiliated Hospital, Fujian Medical University, Fuzhou, Fujian, China”, it should be “Fujian Provincial Institutes of Brain Disorders and Brain Sciences, First Affiliated Hospital, Fujian Medical University, Fuzhou, Fujian, China.”

In the published article, there was an error regarding the affiliations for Zijian Zheng, Qianxi Chen, Zhicheng Wang, Jun Fu, Wenjian Fan, Yuanxiang Lin, Changzhen Jiang, Zhangya Lin. As well as having affiliation(s) 1, they should also have “Department of Neurosurgery, Neurosurgery Research Institute, The First Affiliated Hospital, Fujian Medical University, Fuzhou, Fujian, China.”

There was an error regarding the affiliations for Dezhi Kang, Xiaorong Yan. As well as having affiliation(s) 1,4, they should also have “Department of Neurosurgery, Neurosurgery Research Institute, The First Affiliated Hospital, Fujian Medical University, Fuzhou, Fujian, China.”

The authors apologize for these errors and state that this does not change the scientific conclusions of the article in any way. The original article has been updated.

