# Supplementary material for: A clinical practical model for preoperative prediction of visual outcome for pituitary adenoma patients in a retrospective and prospective study
Source: Front Endocrinol (Lausanne). 2024 Dec 13;15:1479442. doi: 10.3389/fendo.2024.1479442 (PMC11671264; doi:10.3389/fendo.2024.1479442)
Supplement: Supplementary file 1 [file Table1.docx]

As visual impairments caused by adenoma lead to a wide range of functional problems in the daily life, clinical measures of visual acuity do not sufficiently reflect the overall visual function in those with adenoma. Self-reported measures of vision-related quality of life allow one to examine a patient’s subjective well-being and visual disability while performing daily tasks. The VF-14 scale was constructed by Steinberg et al. as an index of functional impairment designed to serve a patient-reported outcome measure, originally for cataract patients.^[1]^

Before ETS and approximately 1 week after surgery,all patients were completed VF-14. At this time, patients also were asked a transitional question about their improvement with regard to their vision after ETS,“How is your vision now compared with how it was before your surgery?” with 5 answer options ranging from “a great deal better” to “a great deal worse.”

The MCID was estimated for VF-14 domains, by the mean change score for patients whose response to the transitional question was “somewhat better”.For the comparison,variables were compared using non-parametric tests.


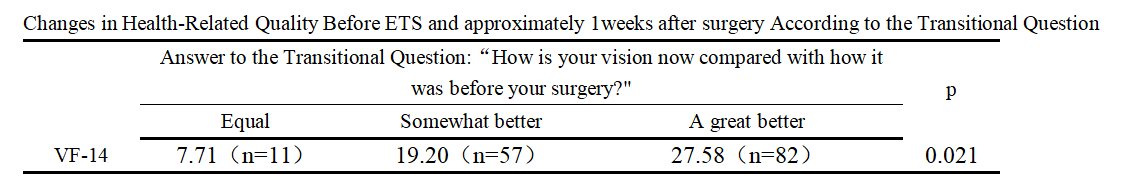


The subgroup denoted by “equal” combined those patients who answered “somewhat worse,” or “a great deal worse” to the transitional question because the sample size of those was very small.

In the end, we identify that the minimal clinically important differences (MCID) after surgery were 19.20 for VF-14 results.We believe that when the difference between preoperative and postoperative scores is 19.20 or above, the visual function of patients is improved after surgery.

1. Steinberg EP, Tielsch JM, Schein OD, Javitt JC, Sharkey P, Cassard SD, Legro MW, Diener-West M, Bass EB, Damiano AM, et al. The VF-14. An index of functional impairment in patients with cataract. Arch Ophthalmol. 1994 May;112(5):630-8. doi: 10.1001/archopht.1994.01090170074026. PMID: 8185520.
